# Kenya’s response to the COVID-19 pandemic: a balance between minimising morbidity and adverse economic impact

**DOI:** 10.12688/aasopenres.13156.2

**Published:** 2021-03-29

**Authors:** Edwin N. Wangari, Peter Gichuki, Angelyne A. Abuor, Jacqueline Wambui, Stephen O. Okeyo, Henry T.N. Oyatsi, Shadrack Odikara, Benard W. Kulohoma

**Affiliations:** 1 Centre for Biotechnology and Bioinformatics, University of Nairobi, Nairobi, Kenya

**Keywords:** Kenya, COVID-19, pandemic response, transmission, disease control measures

## Abstract

Coronavirus disease 2019 (COVID-19) has ravaged the world’s socioeconomic systems forcing many governments across the globe to implement unprecedented stringent mitigation measures to restrain its rapid spread and adverse effects. A disproportionate number of COVID-19 related morbidities and mortalities were predicted to occur in Africa. However, Africa still has a lower than predicted number of cases, 4% of the global pandemic burden. In this open letter, we highlight some of the early stringent countermeasures implemented in Kenya, a sub-Saharan African country, to avert the severe effects of the COVID-19 pandemic. These mitigation measures strike a balance between minimising COVID-19 associated morbidity and fatalities and its adverse economic impact, and taken together have significantly dampened the pandemic’s impact on Kenya’s populace.

## Disclaimer

The views expressed in this article are those of the authors. Publication in AAS Open Research does not imply endorsement by the AAS.

The world is experiencing a significant public health threat due to the global coronavirus disease 2019 (COVID-19) pandemic. The COVID-19 pandemic overshadows recent outbreaks of severe acute respiratory syndromes (SARS) and Middle East respiratory syndrome (MERS) in 2003 and 2012 respectively, which are also caused by viruses that are closely related genetically ^1^. The COVID-19 pandemic has forced all affected countries to adopt drastic response measures, which included imposing total lockdown of cities and even countries, due to its rapid person-to-person transmission rates ^2, 3^. This pandemic is now established in all 54 African countries and coincides with other humanitarian emergencies ^4^. Although Africa still bears a small proportion (4%) of the global pandemic morbidity burden, the WHO forewarns that if left unchecked, COVID-19 could result in nearly a quarter of a billion morbidities, and 150,000 fatalities within a year ^5^. Scientists still remain puzzled by why the pandemic seems to have “spared” Africa, which has fragile healthcare systems ^6– 8^. Several hypotheses have been advanced to explain this occurrence, that include: warmer climate that does not favour the viral pathogen viability, fewer COVID-19 associated deaths because of a comparatively younger population, lower case numbers due to inadequate testing, population-wide immune priming due to previous exposure to other infectious diseases, and genetic factors that protect Africans from severe disease ^6, 7, 9^. The relative contribution of these factors still remains unknown. We opine that it is Africa’s previous experience with life-threatening infectious disease outbreaks, for example Ebola, HIV, and malaria that led to an overreaction by African states to implement a raft of stringent countermeasures to protect their healthcare systems from being overwhelmed. Governments in African countries moved with commendable speed to implement countermeasures at early stages of COVID-19 detection within their borders to restrain widespread disease and its adverse effects ^10^. However, these control measures are thought to be unsustainable and projected to have negative and inequitable impacts in resource poor settings ^11^. In this open letter, we highlight some of the countermeasures against COVID-19 transmission in Kenya, which intend to strike a balance between minimising COVID-19 associated morbidity and fatalities and its adverse economic impact.

Physical distancing minimises person-to-person COVID-19 transmission in a population. This protects individuals at greatest risk of presenting poor infection outcomes from both symptomatic and asymptomatic infected individuals, thereby restricting an increase in the basic reproduction number ( *R*_0_) ^11, 12^. *R*_0_ is a proxy measure of pathogen transmissibility representing the number of individuals infected by a single infected individual in a population, and higher values indicate increasing transmissibility ^13, 14^. In Kenya, the government imposed a nationwide dawn to dusk curfew; and restricted movement within urban areas with high COVID-19 transmission rates, as well as, into and out of urban cities with COVID-19 incidences to rural areas with lower incidence rates. The majority of the older demographic reside in rural areas; and this effort restricted mass migrations to rural areas as a result of economic distress in urban settings, leading to consequent infection of the elderly, and more vulnerable minorities ^15^. Learning institutions and day-care centres were closed. Workplaces that do not provide essential services were advised to allow individuals to work from home; and effect physical distancing measures in the event that workers were absolutely required to access their workstations. All mass gatherings, faith-based events, festivals, conferences and meetings, trade fairs, sporting and cultural events were prohibited to minimise person-to-person contact. The rationale was that it was challenging to maintain physical distance for large crowds, for example at the exit and entrance spaces or even in public transportation ^15^. However, the success of these countermeasures requires implementation over an extended period. Social contact with colleagues, family, and friends via digital media, for example over the phone or Internet, encouraged adherence to physical distancing. The government launched a network of giant internet-enabled balloons in-conjunction with Google to deliver emergency Internet across the country ^16^. This Internet connection was also used for e-learning, working from home, and fostered e-commerce.

Good personal hygiene and sanitization measures are critical for COVID-19 control. Kenya launched nation-wide media campaigns to educate the citizens on the proper handwashing techniques and use of face masks immediately after the first COVID-19 case was detected in March 2020. These campaigns recommend use of soap and running water, 70% alcohol-based sanitizer or 0.1% sodium hypochlorite to wash hands and clean surfaces. Local artisans in the informal business sector were given financial support to assemble handwashing stations and sanitization equipment using available raw materials and re-cycled parts that could be rapidly distributed for use across the country. Fumigation of infection hotspots, for example markets, public transport, and hospitals, was performed routinely. Handwashing was also mandatory prior to entry of any public premises and before boarding public transportation. In Kenya, personal protective equipment (PPE) was primarily imported prior to the COVID-19 pandemic. However, their necessity and shortfall during the pandemic provided an opportunity of capacity and technological leapfrogging, and PPEs are now manufactured locally, and also exported to the wider East African Region. The ministry of health and healthcare stakeholders developed new protocols and policies on handling of deceased remains and conducting funerals. For example, funerals were restricted to a maximum of 15 attendees practicing physical distancing ^17^. Civil society organisations and the government joined efforts to restrict widespread COVID-19 disease by providing personal protective equipment such as face masks, gloves, sanitisers, medical supplies, soap, as well as water and food rations to affected informal settlements across Kenya ^18^. Community social workers were also deployed to raise awareness to the public and educate them on physical distancing and handwashing, COVID-19 prevention control measures, and psychosocial support to affected communities ^18^.

Comprehensive surveillance and detection systems enable data collation and analyses to establish COVID-19 transmission dynamics and societal impact. These systems should enable control at three levels ^19^: (i) First, enable rapid detection, isolation, testing and management of suspected cases. (ii) Secondly, guide the implementation of control measures and be able to contain outbreaks among vulnerable populations, monitor long-term epidemiologic trends and evolution of SARS-CoV-2 virus, and evaluate the impact of the pandemic on the healthcare system. (iii) Finally, incorporate capacity sufficient for understanding the co-circulation of SARS-Cov-2 virus and other respiratory viruses. These systems provide robust evidence used for developing implementation policies required for disease management ^20^. In Kenya the integrated disease surveillance and response (IDSR) system guides the rapid detection, reporting, management and treatment of the reported infection cases ^21^. Seroprevalence and genomic studies provide estimates for the level of COVID-19 infection cases across Kenya, and determine genomic diversity of strains in circulation ^22– 24^. In addition, sentinel surveillance of influenza-like illness and other acute respiratory infections using the global influenza surveillance and response system (GISRS) has allowed robust monitoring of community transmission of COVID-19, and provides insight on co-circulation of respiratory viruses ^25^. Consequently, this has informed more robust and customised public health responses. As part of the East African community response unit (EARCC), Kenya and other partner states continue improve the region’s response capacity on disease prevention, safety and surveillance at border points ^26^.

The Ministry of Health communicates daily via all media outlets the number of confirmed cases, fatalities, recoveries, overall COVID-19 related bed occupancy in various hospitals, and the prevalence in all 47 counties. They also remind all citizens to continue taking precautions not to contract COVID-19; and provide contact details on how and where to seek assistance if you present symptoms. In addition the government has established the COVID-19 risk communication and community engagement sub-committee, in conjunction with media agencies, healthcare stakeholders and the International Organization for Migration (IOM) to enhance strategic communication and community engagement, promote trust and influence risk perception ^27– 30^. Community health workers were also deployed to provide mental health and social support, for example managing loss or grief ^31^.

Mitigation measures that minimise COVID-19 associated morbidity and fatalities have resulted in economic losses and a decline in global economic activity. For example, the tourism and hospitality industry a major foreign exchange earner for Kenya suffered huge losses due to global restriction of movement. Kenya National Bureau of Statistics (KNBS) estimates that up to 1.7 million Kenyans across all sectors lost employment between March and May of 2020 ^32^.

The government unveiled an 8-point economic stimulus program incorporated in the national budget to stimulate economic activity and safeguard livelihoods ^33^. Examples include: A cash transfer programme targeting the elderly, poor and vulnerable was implemented to safeguard the dignity and welfare of the most severely affected. Hiring of ten thousand teachers and purchase of locally fabricated school equipment to support digital learning. Provision of seed capital to small and medium enterprises through a credit guarantee scheme, as well as fast-tracking of tax refunds and other pending payments. Duty remission on raw materials used for domestic manufacturing was implemented. Hiring of additional healthcare workers and expansion of bed capacity in public hospitals. Supply of farm inputs to small scale farmers through an e-voucher scheme. Provision of soft loans to hotels and related establishments. Rehabilitation of wells, water pans and underground tanks in arid and semi-arid areas and flood control measures to protect communities from adverse environmental effects during the COVID-19 pandemic. Enforcing policies that support the purchase of locally manufactured products. A post COVID-19 economic recovery strategy was formulated to dampen the adverse economic effects and reposition the economy on a steady and sustainable growth trajectory ^34^. We posit that more effort should be directed towards achieving a delicate balance between minimising COVID-19 associated morbidity and preventing an economic recession, which is paramount to avoid reversing the gains made on the Sustainable Development Goals. We conclude that overall, these response measures together with others have significantly dampened the pandemic’s impact on Kenya’s populace.

## Data availability

### Underlying data

No data are associated with this article. 
