# Supplementary material for: Kenya’s response to the COVID-19 pandemic: a balance between minimising morbidity and adverse economic impact
Source: AAS Open Res. 2021 Mar 29;4:3. Originally published 2021 Jan 14. [Version 2] doi: 10.12688/aasopenres.13156.2 (PMC7921885; doi:10.12688/aasopenres.13156.2)
Supplement: Peer review history

# Peer review: Reviewer response for version 2

**DOI:** 10.21956/aasopenres.14329.r28533

**Published:** 2021-05-17

**Authors:** Gisele Umviligihozo, Francis Mwimanzi

**Affiliations:** 1 Faculty of Health Sciences, Simon Fraser University, Burnaby, BC, Canada

Edwin N. Wangari and colleagues adequately revised the article according to our comments and suggestions, we are satisfied with their response and have no further comments.

 We noticed that the authors’ response, reffered to article number 28 as one addressing the adverse economic impact of SARS-CoV-2 on Kenyan’s livelihoods while the correct reference article number is 32, we suggest to correct this in the author's response for accuracy.

Does the article adequately reference differing views and opinions?

Partly

Are all factual statements correct, and are statements and arguments made adequately supported by citations?

Yes

Is the Open Letter written in accessible language?

Yes

Where applicable, are recommendations and next steps explained clearly for others to follow?

Not applicable

Is the rationale for the Open Letter provided in sufficient detail?

Yes

Reviewer Expertise:

Virology, Immunology

We confirm that we have read this submission and believe that we have an appropriate level of expertise to confirm that it is of an acceptable scientific standard.

# Peer review: Reviewer response for version 1

**DOI:** 10.21956/aasopenres.14259.r28383

**Published:** 2021-03-01

**Authors:** Brian Godman

**Affiliations:** 1 School of Pharmacy, Sefako Makgatho Health Sciences University, Pretoria, South Africa; 2 Strathclyde Institute of Pharmacy and Biomedical Sciences, Glasgow, UK

Thank you - I enjoyed reading this Open letter. However, I have a number of concerns. Firstly, there is a lack of documentation of activities in Kenya and also how these relate to other African countries to provide future direction. A paper I was involved with, Ogunleye *et al.,* with co-authors from Kenya, extensively documented both healthcare and financial activities to help prevent the spread of the virus across Africa and the implications going forward. It would be good to add to this for Kenya, building on the comments made - especially with Africa learning from other infectious diseases in Ogunleye OO *et al. *(2020) ^1^ *.*

 There have also been concerns with the misinformation regarding COVID-19 and treatments - especially important in countries with high co-payment levels such as Kenya - as discussed in a paper of mine, Sefah I *et al. *(2020) ^2^. Encouragingly, in Kenya there is limited/no self-purchasing of antimicrobials unlike a number of other African countries. We are also seeing African countries innovate, etc., to help with the pandemic - providing opportunities for the future demonstrated in another paper I was involved in, Afriyie DK *et al.* (2020) ^3^. It would be good to build on this for Kenya, along with potential plans about greater local production of medicines, to reduce issues of shortages in the future and help with the economy going forward.

Does the article adequately reference differing views and opinions?

Yes

Are all factual statements correct, and are statements and arguments made adequately supported by citations?

Yes

Is the Open Letter written in accessible language?

Yes

Where applicable, are recommendations and next steps explained clearly for others to follow?

Partly

Is the rationale for the Open Letter provided in sufficient detail?

No

Reviewer Expertise:

My area of research includes both infectious and non-infectious diseases across Africa and wider.

I confirm that I have read this submission and believe that I have an appropriate level of expertise to confirm that it is of an acceptable scientific standard, however I have significant reservations, as outlined above.

## References

[1] : Response to the Novel Corona Virus (COVID-19) Pandemic Across Africa: Successes, Challenges, and Implications for the Future. *Front Pharmacol*.2020;11: 10.3389/fphar.2020.01205 1205 10.3389/fphar.2020.01205 33071775PMC7533592

[2] : Rapid Assessment of the Potential Paucity and Price Increases for Suggested Medicines and Protection Equipment for COVID-19 Across Developing Countries With a Particular Focus on Africa and the Implications. *Front Pharmacol*.2020;11: 10.3389/fphar.2020.588106 588106 10.3389/fphar.2020.588106 33628173PMC7898676

[3] : COVID-19 pandemic in resource-poor countries: challenges, experiences and opportunities in Ghana. *J Infect Dev Ctries*.2020;14(8) : 10.3855/jidc.12909 838-843 10.3855/jidc.12909 32903226

# Response

**Published:** 2021-03-13

**Authors:** Benard Kulohoma

We would like to thank the reviewer for their constructive comments that have strengthened our manuscript. The aim of which was to highlight countermeasures taken in Kenya. A separate article describing countermeasures taken across Africa by the authors has already been described in the text (Reference 3).

# Peer review: Reviewer response for version 1

**DOI:** 10.21956/aasopenres.14259.r28411

**Published:** 2021-03-01

**Authors:** Peter Macharia

**Affiliations:** 1 Population Health Unit, KEMRI Wellcome Trust Research Programme, Nairobi, Kenya

The article summarises measures put in Kenya in response to COVID-19. I have a few suggestions on how the manuscript might be improved.

 The authors should be more specific on measures that were put into place. For example, mentioning the major urban areas where movement in/out was restricted, specific areas within Nairobi where the movement was constrained, etc. In its current form, the article is more general and has not been fully localized to the Kenyan context.

 The authors should appreciate that the measures put into place had a temporal and spatial aspect. A chart and/or a map(s) could improve the article by providing a visual of the measures put into place over time in Kenya and citing those that were in specific counties. Also, include the timeline that is being addressed in the article e.g. between March 2020 to February 2021

 The article has not tackled the other side of the coin, the effects of COVID-19 on the economy and how the government of Kenya and other stakeholders responded to minimize the effects.

 The article mentions the value seroprevalence, genomic and sentinel surveillance studies had on Kenya. These are just but a few themes. Consider adding other lines such as disease mapping, economic modelling, gender-related studies among others

 The message captured by the title has not been substantiated in the article. The balance between minimizing morbidity and adverse economic impact has not been elucidated in the Kenyan context

 Provide references to sources of the measures cited

Does the article adequately reference differing views and opinions?

No

Are all factual statements correct, and are statements and arguments made adequately supported by citations?

Partly

Is the Open Letter written in accessible language?

Yes

Where applicable, are recommendations and next steps explained clearly for others to follow?

Not applicable

Is the rationale for the Open Letter provided in sufficient detail?

Partly

Reviewer Expertise:

Spatial epidemiology/ health geography

I confirm that I have read this submission and believe that I have an appropriate level of expertise to confirm that it is of an acceptable scientific standard, however I have significant reservations, as outlined above.

# Response

**Published:** 2021-03-13

**Authors:** Benard Kulohoma

We would like to thank the reviewer for their constructive comments that have strengthened our manuscript. We have revised the manuscript to make it more clear that movement was also restricted within urban areas with high rates of COVID-19 transmission. The concise Open Letter article is intended to give a general perspective of the countermeasures taken across Kenya. It would be beyond the scope of the article to describe countermeasures taken in each County or administrative unit in Kenya. We did not perform temporal and spatial analyses and therefore do not present data or findings on the same. We have described the adverse effects of COVID-19 on the economy giving an example of the tourism sector, which is one of the major contributors to Kenya’s GDP, and highlighted that the government has implemented a detailed 8-point economic stimulus program incorporated in the national budget to stimulate economic activity and safeguard livelihoods (Reference 28). In addition we now provide more examples. We have revised the manuscript to highlight societal impacts, for example the loss of jobs across all sectors affecting up to 1.7 million Kenyans, and provide additional references, with more details.

# Peer review: Reviewer response for version 1

**DOI:** 10.21956/aasopenres.14259.r28388

**Published:** 2021-02-24

**Authors:** Peninah Muthoni Wairagu

**Affiliations:** 1 Technical University of Kenya, Nairobi, Kenya

The open letter highlights the mitigation measures employed by the Kenyan government to minimize the COVID-19-related morbidities and mortalities in Kenya and their impact on the economy.

 Comments on the letter include;

1. The title of the letter suggests that the authors have analyzed the mitigation measures employed and their economic impact. However, the content of the letter is skewed towards the mitigation measures employed and does not adequately discuss the economic impact of these measures. There is only a brief mention on the effect on tourism, which only forms a part of the Kenyan economy. The mitigation measures also had effects on other sectors of the economy, such as employment, export and import business, manufacturing among others. It is also important for the authors to highlight that not all of the impact was negative, as seen in the manufacture of personal protective equipment (PPEs), soaps and sanitizers as well as service industries such as internet services provision.
2. The use of PPEs was a major aspect of the mitigation measures employed by the government to fight COVID-19, yet it is not mentioned in the open letter. It is important for the authors to include a discussion on PPEs especially because their manufacture locally had positive impact on the economy.
3. Part of the reason why Africa was expected to bear the greater burden of COVID-19 was due to the poor health care systems including the lack of enough hospitals and healthcare workers. The government sought to mitigate this by hiring health workers and some counties built hospitals. A discussion on this can also be included to make the letter more comprehensive.
4. The authors need to give a brief description on reproduction number (R _o_) since it is not common knowledge.

Does the article adequately reference differing views and opinions?

Partly

Are all factual statements correct, and are statements and arguments made adequately supported by citations?

Yes

Is the Open Letter written in accessible language?

Yes

Where applicable, are recommendations and next steps explained clearly for others to follow?

Not applicable

Is the rationale for the Open Letter provided in sufficient detail?

Yes

Reviewer Expertise:

Cancer

I confirm that I have read this submission and believe that I have an appropriate level of expertise to confirm that it is of an acceptable scientific standard.

# Response

**Published:** 2021-03-13

**Authors:** Benard Kulohoma

We would like to thank the reviewer for their constructive comments that have strengthened our manuscript. We have now provided examples of these measures in the 8-point economic stimulus program (Reference 28). We have revised the manuscript to highlight the manufacture, distribution and use of PPEs is restricting COVID-19. Capacity building to strengthen healthcare systems across Africa has already been highlighted at detail by the authors in a different article (Reference 3). We have also made revisions to the manuscript to give a brief description of reproduction number (R _o_).

# Peer review: Reviewer response for version 1

**DOI:** 10.21956/aasopenres.14259.r28384

**Published:** 2021-02-24

**Authors:** Gisele Umviligihozo, Francis Mwimanzi

**Affiliations:** 1 Faculty of Health Sciences, Simon Fraser University, Burnaby, BC, Canada

I read with interest the article authored by Edwin N. Wangari and colleagues that presented Kenya’s response to the COVID-19 pandemic: a balance between minimizing COVID-19 morbidity and the adverse economic impact.

 The article dealt with an important topic that deserves to be accepted. I have minor suggestions for improvement.

 This open letter clearly detailed the Kenya’s COVID-19 response and its impact on reduction of COVID-19 morbidity and spread, however it lacks sufficient supporting information on the economic component. The authors noted that mitigation measures that minimize COVID-19 associated with morbidity and fatalities have resulted in losses and a decline in a global economic activity but only mentioned an impact on the tourism and hospitality industry in Kenya. The article would gain strength by providing more detailed information on the negative economic impact of the COVID-19 response on other national economic activities and international trade that mainly contribute to the general livelihoods of Kenyans, such as agriculture, livestock, fishing and transport industries as well as small businesses (local markets, bars, restaurants, fashion houses and beauty salons).  

 To further substantiate the argument, the authors could highlight critical points regarding the balance between the response to COVID-19 and its impact on the declining economy that is lacking in the government’s economic stimulus packages.

Does the article adequately reference differing views and opinions?

Partly

Are all factual statements correct, and are statements and arguments made adequately supported by citations?

Yes

Is the Open Letter written in accessible language?

Yes

Where applicable, are recommendations and next steps explained clearly for others to follow?

Not applicable

Is the rationale for the Open Letter provided in sufficient detail?

Yes

Reviewer Expertise:

Virology, Immunology

We confirm that we have read this submission and believe that we have an appropriate level of expertise to confirm that it is of an acceptable scientific standard, however we have significant reservations, as outlined above.

# Response

**Published:** 2021-03-13

**Authors:** Benard Kulohoma

We would like to thank the reviewer for their constructive comments that have strengthened our manuscript. It is beyond the scope of the concise. We have now provided examples of these measures in the 8-point economic stimulus program (Reference 28).
